# Spun of improvised *cis*‐1,3,4,6‐tetranitrooctahydroimidazo‐[4,5‐d]-Imidazole (BCHMX) in polystyrene nanofibrous membrane by electrospinning techniques

**DOI:** 10.1186/s13065-022-00853-7

**Published:** 2022-08-09

**Authors:** Ahmed K. Hussein, Ahmed Elbeih, Mohamed Mokhtar, Mahmoud Abdelhafiz

**Affiliations:** Military Technical College, Kobry Elkobbah, Cairo, Egypt

**Keywords:** Electrospun nanofibers, Electrospinning, BCHMX, Polystyrene, Thermal study, Sensitivities

## Abstract

Development of ultra-fine fiber technology and nano-sized materials are widely taking place to enhance the characteristic of different materials. In our study, a newly developed technique was used to produce improvised nano energetic fibers with the exploitation of *cis*‐1,3,4,6‐Tetranitrooctahydroimidazo‐[4,5‐d] imidazole (BCHMX) to spin in a polystyrene nanofiber membrane. Scanning electron microscopy (SEM) showed the synthesized nanofibrous polystyrene (PS)/BCHMX sheets with clear and continual fiber were imaged with scanning electron microscopy (SEM). Characterization of the produced nanofiber was examined by Fourier Transform Infrared (FTIR), and X-ray diffractometer (XRD). Explosive sensitivity was also evaluated by both BAM impact and friction apparatus. Thermal behavior for the synthesized PS/BCHMX fiber and the pure materials were also investigated by thermal gravimetric analysis (TGA). The results show enhancement in the fabrication of nano energetic fibers with a size of 200–460 nm. The TG confirms the high weight percentage of BCHMX which reaches 60% of the total mass. PS/BCHMX fiber was confirmed with the XRD, FTIR spectrum. Interestingly, XRD sharp peaks showed the conversion of amorphous PS via electrospinning into crystalline shape regarding the applied high voltage. The synthesized PS/BCHMX nanofiber was considered insensitive to the mechanical external stimuli; more than 100 J impact energy and  > 360 N initiation force as friction stimuli. PS/BCHMX is considering a candidate tool to deal with highly sensitive explosives safely and securely for explosives detection training purposes.

## Introduction

Electrospinning is a novel entry in nanomaterial applications that has been exploited recently on a wide range to fabricate composite polymeric nanofiber membranes simply and straightforwardly [1–4]. These polymeric nanofibers have acquired great attention through their diverse applications as biomaterials, biomedical additives for wounds and burns dressings, drug delivery, and sensors [5–8]. Electrospun technique has been used to perform nano-fiber eletrodes for detection of bacterial agent [9] Electrospinning can produce nanofiber which expects to possess an extremely high surface area and a defect-free crystalline structure [10, 11]. The obtained polymeric nanofibrous membranes should be characterized through the polymeric features, processing parameters, and the surrounding environmental factors [12–15].

*Cis*‐1, 3, 4, 6‐tetranitrooctahydroimidazo‐[4,5‐d] imidazole, BCHMX/bicyclo-HMX, is an interesting novel explosive substance with distinctive explosive characteristics. It could be synthesized through a two-step approach [16, 17]. BCHMX is white crystalline explosive, it has 224 °C ignition temperature and it possesses a high performance together with relatively high sensitivity in comparison with pentaerythritol tetranitrate (PETN) [18–21]. Different publications focus on reducing the sensitivity of BCHMX by using a different polymeric matrix such as Viton A 200 [19, 21, 22], poly dimethyl siloxanes (PDMS) [19, 21], acrylonitrile-butadiene rubber (NBR) [19, 21, 23], polyisobutylene (PIB) [21, 24] and softened poly-(methyl methacrylate) (PMMA) [24]. Several nitramines explosives have been studied in comparison with BCHMX. The penetration performance in shaped charges were studied [25]. The detection of BCHMX was published in literature [26]. The thermal study and kinetic parameters of BCHMX in mixture with insensitive explosives such as FOX-7, NTO and TNT were also considered [27–30]. In addition, the study of advanced new energy materials and BCHMX in the rocket propellant mixtures were presented in literature [31–34].

However, BCHMX applications are still restricted regarding their potential characteristics, thus grabbing countless investigations. Herein, electrospinning was used to electrospun polystyrene PS/BCHMX solution to fabricate nanofibrous PS/BCHMX membranes. In this work, the electrospinning governed parameters; concentration, functional voltage, feed rate, needle to drum distance, rotating mandrel speed, and time [35], were firstly optimized to ensure the fabrication of continuous PS/BCHMX nanofibers. Then, the fabricated nanofibrous mats were characterized via FTIR, SEM, and XRD. The thermal behavior of PS/BCHMX was investigated through Thermal Gravimetric Analysis (TGA), while the sensitivity of these nanofibers towards impact and friction effects were determined experimentally using the BAM instruments with the probit analysis method.

## Experimental

### Materials

**Fig. 1:**
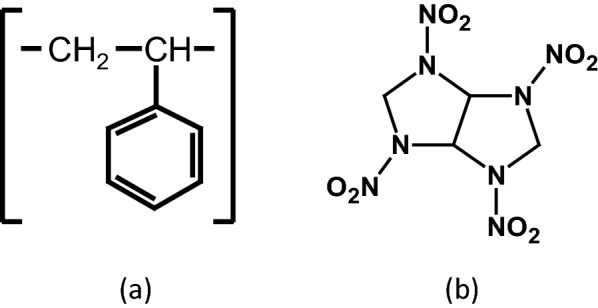
Structural formulas of **a** Polystyrene polymer, **b** BCHMX

Polystyrene (PS), Fig. 1a, [M_w_ = 45,000 g/mol] was supplied from Al-Gomhoria Company for medicines and medical supplies, Egypt. BCHMX was synthesized locally following the methodology published in [16, 17] and its chemical structure is given in Fig. 1b. Dimethyl Formamide (DMF) and chloroform were obtained from Sigma-Aldrich, Germany, and were used as solvents for BCHMX and PS. All acquired chemicals were used in the experimental work as received without any further purification.

### Preparation of PS/BCHMX nanofiber via electrospinning

The proper fabrication of PS/BCHMX nanofibrous membranes via the electrospinning technique was performed through a specifically designed strategy as follows; a DMF-chloroform mixture was firstly prepared with a volumetric ratio of 1:3. Then, BCHMX crystals together with PS particles were added to the prepared.

DMF-chloroform mixture at 60% w/w ratio. The mixture was sonicated for 2 h and then electrospun at a voltage of 18.5 kV, for 3 h continuously. The needle-collector distance was adjusted at 14 cm, while the feed rate and the speed of the rotating mandrel were adjusted at 20 µL/min and 500 rpm respectively. This strategy helps to overcome the technical challenges as well as optimize all the factors affecting the whole process; solution viscosity, solids concentration, flow rate, applied voltage, the distance between syringe needle and collector, collector rotating speed, and the time of electrospinning session. Firstly, the solvent used to dissolve PS and BCHMX was selected carefully to ensure the formation of a continuous solution jet from the needle tip. Different solvents and various solvent mixtures have been tried with a wide range of vol/vol % as follows; pure DMF, pure chloroform, DMF-chloroform (1:1), (2:1), (3:1), (4:1), (1:2), (1:3), and (1:4) vol/vol %. Afterward, 60% BCHMX (w/w% concerning polystyrene) was added to PS/solvent mixture forming the maximum packing density of BCHMX that could form a homogenous PS/BCHMX suspension. This homogeneity together with the overall viscosity of the mixture were critical issues to avoid the formation of undesirable separate drops or blockage of the syringe needle. The good mixing of solids was found to be a golden key to obtaining the appropriate homogeneity. Thus, sonication was a must after the addition of BCHMX particles to the PS solution to obtain a normal distribution of solids through the liquid medium.

**Fig. 2:**
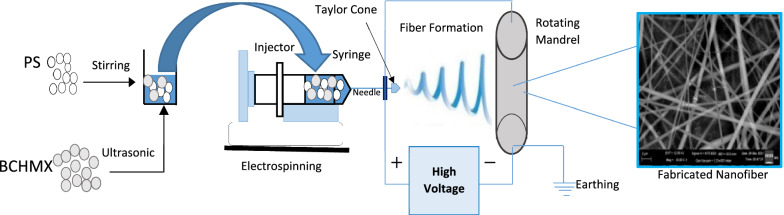
Schematic illustration showing the formation of the characteristic “Taylor Cone” shape prior to the formation of the fibrous jet towards the collector

The PS/BCHMX solution was then subjected to different values of applied voltage as a start of the electrospinning process. The applied voltage greatly affected the obtained jet from the syringe needle towards the collector (rotating mandrel). 5 kV, 8 kV, 10 kV, 12 kV, 15 kV, 18 kV, 20 kV, 22 kV, and 24 kV were used and the shape of the resulting jet was carefully observed. Once the jet coming out from the syringe needle forms a specific shape known as “Taylor Cone” as shown in Fig. 2, this applied voltage is counted as the proper value to electrospun that mixture. The next step was optimizing the flow rate of the solution mixture to guarantee the capability of the obtained jet from the syringe needle to form continuous fiber lines on the collector. Herein, 2 µL/min has been used as a starting flow rate followed by a series of trials at different flow rates; 5 µL/min, 10 µL/min, 13 µL/min, 15 µL/min, 18 µL/min, 20 µL/min, 22 µL/min, 25 µL/min, and 30 µL/min.

At this point, optimizing the distance between the syringe needle and the rotating mandrel was a must to boost the efficiency of the electrospinning of the polymeric mixture. The investigated distances were 12 cm, 14 cm, and 16 cm. It was observed that changing that distance greatly affected the continuity of the fabricated fiber. It was observed that changing the distance between needle and mandrel is greatly connected with the available period for the obtained jet to rotate around the mandrel drum one full round and restart another round continuously without any interruption. During that testing, it was found that optimizing the needle-collector distance should be investigated together with the rotating speed of the collector. Both factors have the same impact on the continuity of the obtained jet. Therefore, an optimization between the distance and mandrel speed was necessary for success. The rotating speed was changed from 300 to 700 rpm at a fixed needle-collector distance. The previous performed efforts were to develop the optimum conditions for the electrospinning of PS/BCHMX solution fabricating a continuous nanofibrous sheet. However, the time of the electrospinning process was the only factor that still has not been investigated at that moment. Herein, the preprepared PS/BCHMX solution (60% w/w ratio respectively) was electrospun at a voltage of 18.5 kV, for 3 h continuously. The needle-collector distance was adjusted at 14 cm, while the feed rate and the speed of the rotating mandrel were adjusted at 20 µL/min and 500 rpm respectively. The concentration of BCHMX was fixed at 60% w/w concerning the polymer concentration.

### Characterization

#### Scanning electron microscopy (SEM)

Electron Scanning Microscopy (SEM, ZEISS SEM EVO 10 MA) was exploited to explore the morphology of the electrospun PS/BCHMX nanofibers. The SEM apparatus is with three detectors; secondary electrons, backscattered electrons, and connected with a Bruker Quantax (200-unit) Energy-Dispersive X-ray spectrometer (EDX) that enables mapping the composition included in every single image. Also, the average diameter of the obtained fibrous PS/BCHMX, PS nanofiber, and pure explosive was determined after image processing for the obtained photos.

#### Fourier transform infrared (FTIR) spectra

Infrared spectra of the pure PS, pure BCHMX samples, and the synthesized PS/BCHMX nanofibers were recorded with a Shimadzu 8000 series FTIR spectrometer. Herein, 2 mg KBr was ground with 350 mg of pure BCHMX and then pressed into light-permeable BCHMX/KBr disks. These disks were then placed in the FTIR instrument for analysis at 500—4000 cm^−1^, 500 scans, and a resolution of 8.0 cm^−1^.

#### X-ray diffraction pattern (XRD)

XRD investigation was accomplished for pure PS (raw material), pure PS nanofibers, pure BCHMX, and PS/BCHMX nanofiber membranes using an X-ray diffractometer, PANalytical Empyreanmeter at 40 kV and 30 mA. Cu tube was provided together with the XRD device and all samples were subjected to a 1.5405 Å irradiation with Cu (K_α_). Finally, XRD diffraction patterns were obtained via recording over the range of 2θ at a temperature range from 15 to 65 °C.

#### Impact and friction sensitivity measurements

The sensitivity of the fabricated PS/BCHMX nanofiber membrane towards impact stimulus was determined in comparison to the pure BCHMX. To do so, dropping adjustable weights, 2 kg and 5 kg, was used for both cases; pure BCHMX and PS/BCHMX nanofibers. This measurement was carried out through the BAM impact sensitivity instrument and the possible initiation limits were determined through the probit analysis method [36, 37]. For each measurement, 50% initiation is considered an adequate initiation of the individual sample. In the same way, the sensitivity towards the friction energy was achieved experimentally. In this measurement, 0.01 g of BCHMX and PS/BCHMX individually was placed on a porcelain plate with extra caution. An adjustable load was used to induce force acting between a pistil and the porcelain plate. Once a characteristic smoke or sound was achieved in at least 50% of tests, the load value is counted as a successful initiation trial. The evaluation was considered a successful initiation with the aid of the probit analysis method [36, 37].

#### Thermal analysis (TGA)

The TGA55, USA was utilized to investigate the thermal decomposition behavior of PS/BCHMX nanofiber membrane, PS nanofiber -membrane, PS polymer, and pure BCHMX. To ensure the accurateness of the attained data, TG mass and pan were calibrated before use. 2 mg of each sample was sited in a high-temperature platinum crucible. Then, the crucible was transferred to the TG sample holder assembly, which had been set at room temperature (around 25 °C). The samples were tested in a temperature range of 40–500 °C, and the whole thermal decomposition process was carried out in an inert atmosphere (N2, 40 ml min^−^1). The experimental data were obtained at a data-collecting rate of 20 points per kelvin. The running experiments with heating rates (β) of 10 K min^−^1.

## Results and discussion

### Optimizing the electrospinning procedure for the preparation of PS/BCHMX nanofibers

**Fig. 3:**
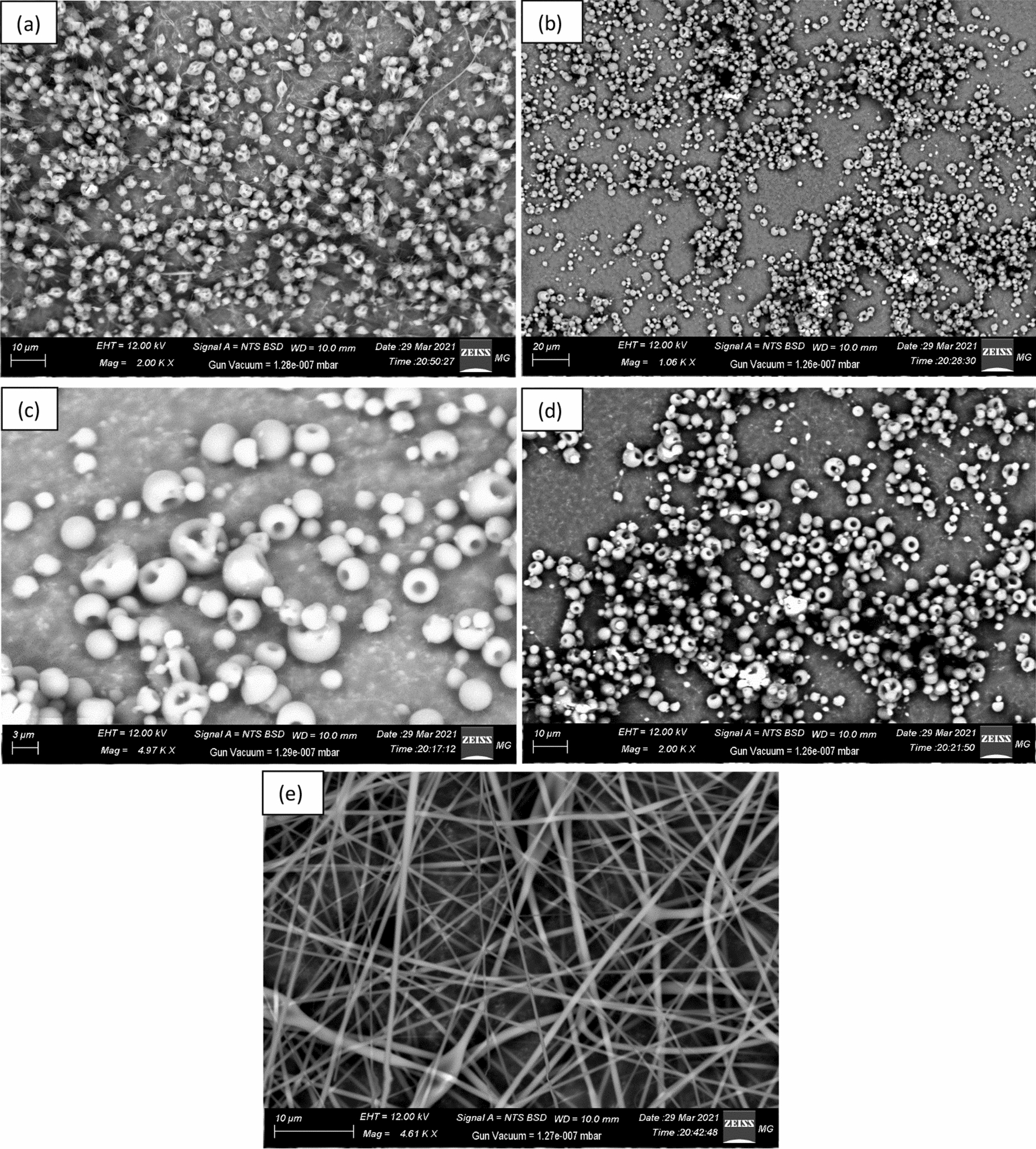
SEM Micrographs for the obtained PS/BCHMX nanofibrous membranes at different processing conditions; **a** 18.5 kV in pure DMF, **b** 18.5 kV in pure Chloroform, **c** 18.5 kV in DMF-Chloroform (3:1), **d** 20.0 kV in DMF-Chloroform (1:3), and **e** 18.5 kV in DMF-Chloroform (1:3)

As discussed previously in the experimental part, it was found that there are too many factors affecting the efficiency of the electrospinning process. However, the viscosity of the polymeric mixture and the applied voltage value have shown a dramatic impact on the whole process. Figure 3 shows the significant difference in the morphology of the collected fibers at different solvents and various applied voltage values respectively. These results confirmed that optimizing these factors has a crucial role in developing an efficient electrospinning process to obtain proper nanofibrous membranes.

### PS/BCHMX nanofiber morphology

SEM micrographs for the electrospun PS/BCHMX show the efficient production of nanofibrous PS/BCHMX composite membrane via electrospinning. Figure 3 shows the formation of continuous nanofibers and successful grafting of BCHMX crystals through the PS frame with an average diameter ranging between 200 and 460 nm. According to Uyar and Besenbacher, the DMF was the best solvent to obtain uniform polystyrene nano-fibers with beads which have average fiber diameter in the range of 443 ± 86 nm [38]. This result proved the compatibility of our results with the literature.

### XRD pattern and FTIR spectrum

**Fig. 4:**
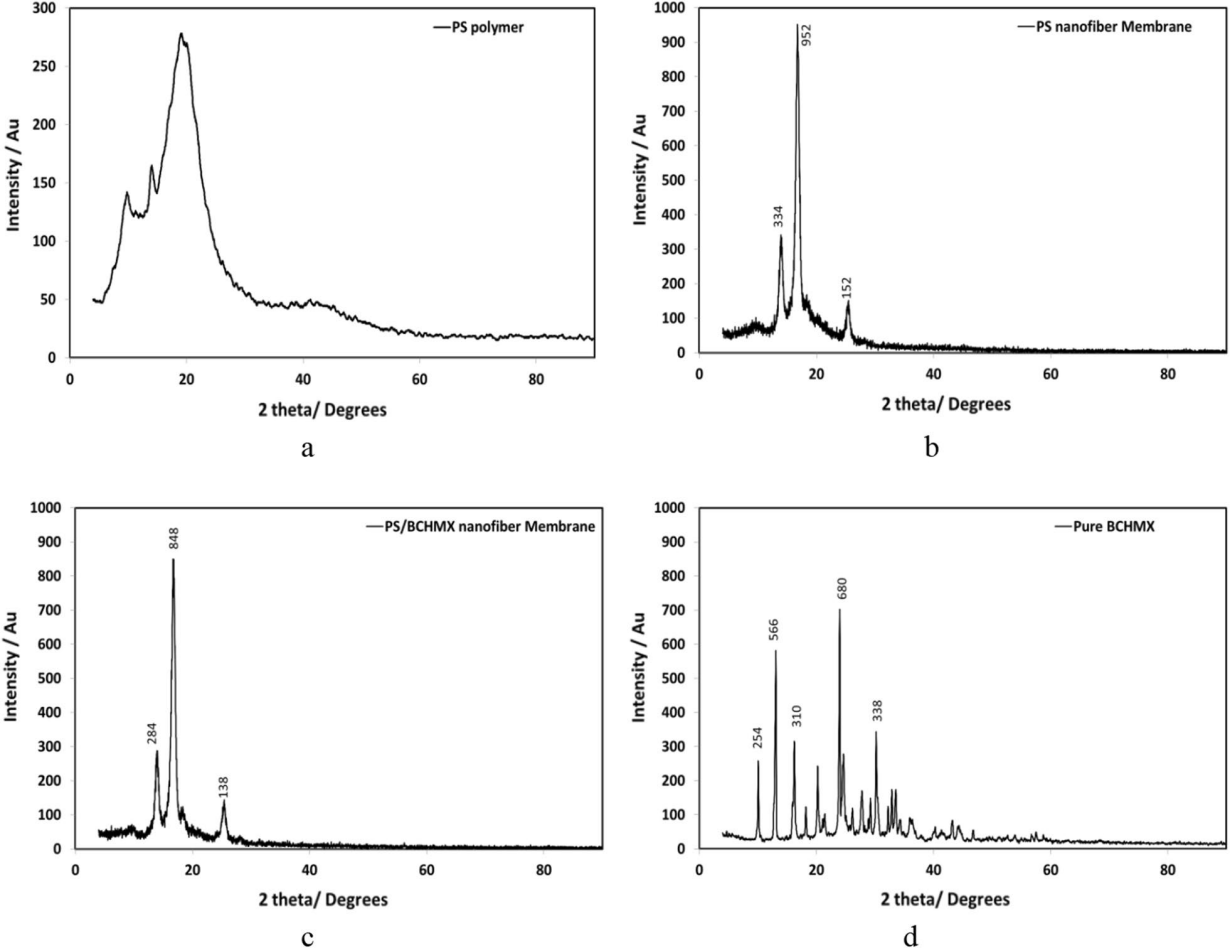
X-ray Diffraction Patterns of PS crystals, **a**. polystyrene Polymer. **b**. PS nanofiber **c**. PS/BCHMX nanofiber membrane **d**. pure BCHMX

Figure 4 illustrates the XRD patterns of polystyrene polymer, PS nanofiber, PS/BCHMX nanofiber membrane, and pure BCHMX. The XRD of amorphous polystyrene polymer before applying the electrospinning against both the electrospun PS and PS/BCHMX nano-fibrous composites. PS raw material has a wide peak at 2θ = 19.06° indicating its basic amorphous nature. On the other hand, electrospun PS fibers indicted a strong sharp peak at 2θ = 16.76°, ascribed to the dramatic change from the amorphous structure into the crystalline form. This indicates that the electrospinning technique is responsible for the significant change in the PS particle structure as it converts the amorphous particles into PS crystals with a high ordering value, indicated by the intensity of the peak of 539. Also, it can be observed that the peaks of electrospun PS nanofiber were shifted when compared with the raw material samples. This is supported by the increase happened in the d-spacing from 4.6 A (PS) to 5.28 A° (submicron-fibrous PS). These results agree with the findings in the literature given in references [39, 40]. The XRD patterns of PS nanofiber and PS/BCHMX nanocomposite membranes after loading BCHMX crystals via the electrospinning process are given in Fig. 4.

**Fig. 5:**
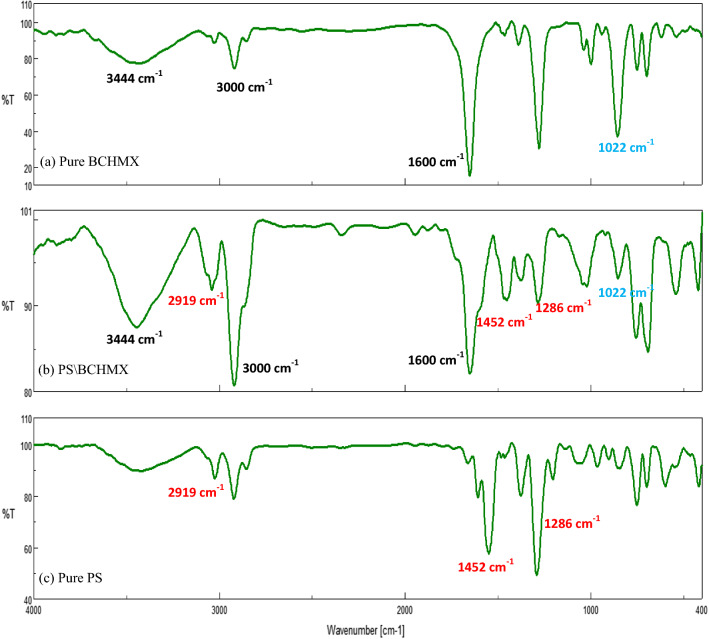
FTIR spectra of **a** Pure BCHMX, **b** PS-BCHMX nano-fibrous Membrane, and **c** Pure PS

As indicated in Fig. 4a and b, the electrospinning technique was able to convert the structure of the PS crystals from an amorphous structure into a highly crystalline one regarding the high voltage applied. It was also observed that PS nanofibers and PS-BCHMX nanofibers have similar peaks at 2θ = 13.959°, 16.76°, and 25.39° however, the peak intensities in the PS nanofibrous peaks, 463, 161, and 65 were increased in the PS-BCHMX nanofibrous membrane content to 539, 372, and 226 respectively. This logical observation could be attributed to the addition of the BCHMX particles to PS crystals. This XRD finding together with the SEM spectrum, given in Fig. 3, and the FTIR spectra, given in Fig. 5, clearly confirms the successful fabrication of PS submicron fibrous membrane loaded with BCHMX explosive crystals.

As it was illustrated, the peak at 3000 cm^−1^ could be assigned to C-H asymmetric stretching vibration in the aromatic-ring structure of BCHMX, while the peak at 1600 cm^−1^ could be attributed to the antisymmetric stretching vibration of C–NO_2_ bonding. Also, the peak at 1388 cm^−1^ could be characteristic of the symmetric stretching of C-NO_2_ bonding. Additionally, the strong absorption peaks detected at 1033 and 744 cm^−1^ could be characteristic peaks assigned to the crystalline structure of BCHMX. On the other hand, the peaks at 3444 cm^−1^ shown in Fig. 5b could be characteristic of the polystyrene structure in the obtained PS/BCHMX nanofibers. These results confirmed the synthesis of PS/BCHMX nanofiber membranes through the electrospinning technique of a solution composed of PS and BCHMX particles dissolved in DMF.

### PS/RDX nanofiber sensitivity to external stimuli

Interestingly, PS/BCHMX nanofibers fabricated via electrospinning showed no impact sensitivity or friction sensitivity to initiate at standard conditions (see Table 1, a sensitivity comparison between pure BCHMX and PS/BCHMX nanofiber). This intense change in the explosive behavior of BCHMX when electrospun with PS could be attributed to the homogenous insertion of BCHMX crystals between the PS nanofibers. The electrospinning technique causes the coverage of BCHMX explosive crystals with a blanket of PS nanofibers which decreases the sensitivity of BCHMX dramatically. Therefore, the formation of the hot spot after exertion to external mechanical stimuli is considered prohibited and difficult to propagate. Consequently, fabricated PS/BCHMX nanofibrous could be considered a candidate tool to deal with highly sensitive explosives safely and securely for training purposes.

**Table 1:** Impact and friction sensitivity for pure BCHMX and PS/BCHMX nanofiber with other plastic explosives

Type	Impact sensitivity (J)	Friction sensitivity (N)
BCHMX-C4 [21]	11.56	181
BCHMX-Form [41]	15.8	228
BCHMX-HTPB [42]	9.6	322
BCHMX-GAP [42]	7.7	294
BCHMX-Sylgard [43]	25.8	246
BCHMX-Viton [21]	5.3	283
BCHMX-FL [21]	5.4	299
BCHMX-TNT [44]	10.8	181
Pure BCHMX [44]	3.2	88
PS/BCHMX	> 100	> 360

**Fig. 6:**
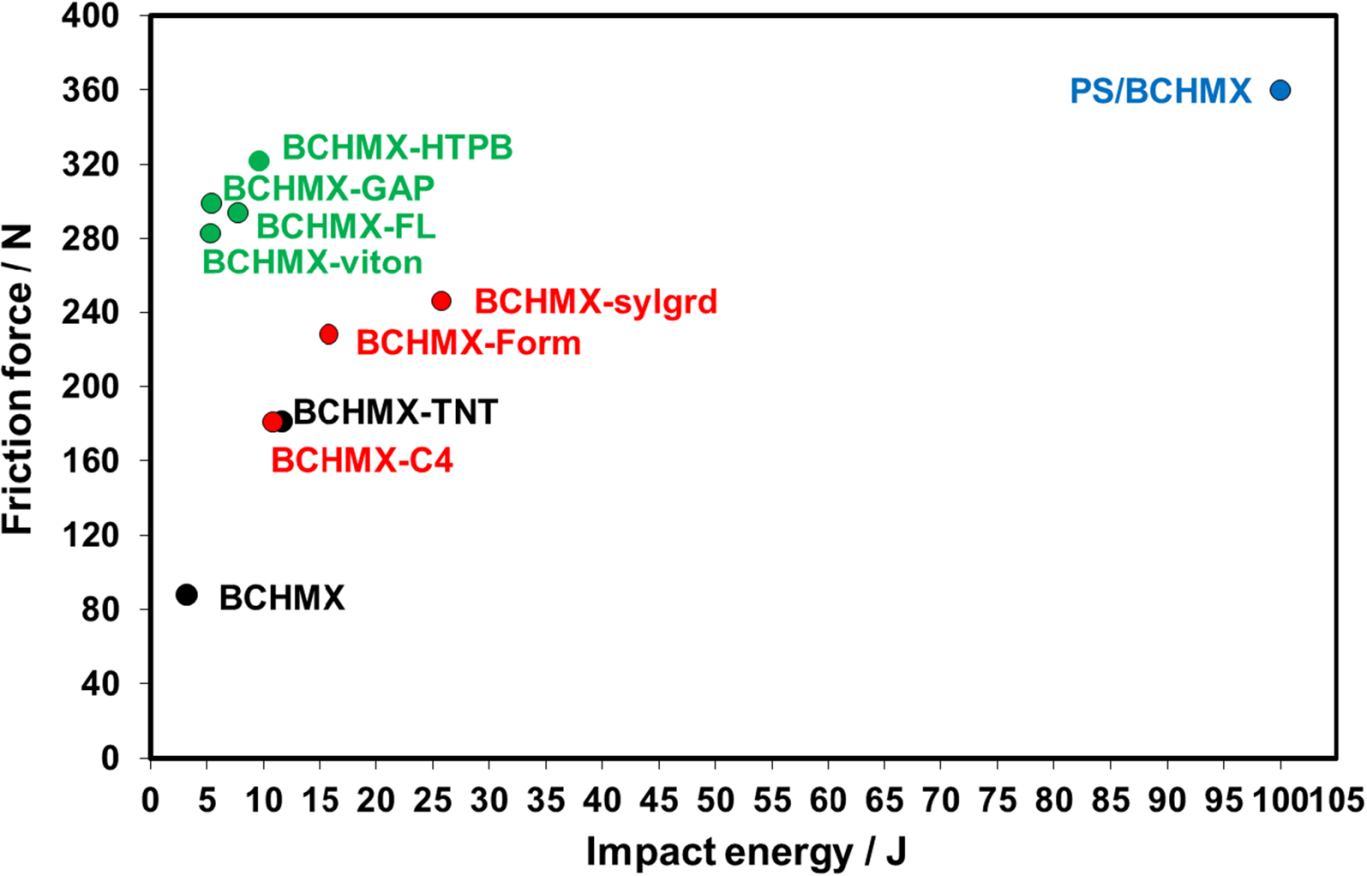
The sensitivities of different explosives compositions based on BCHMX

Figure 6 presents the impact and friction sensitivities of different samples based on BCHMX in comparison with PS/BCHMX. The cast cured samples based on BCHMX with GAP and HTPB binders and the fluorinated BCHMX samples have high impact sensitivity (less than 10 J) while their sensitivities to friction are low. On the other hand, the plastic BCHMX based on elastic matrices (Sylgard, Formex, and C4) have a moderate impact and friction sensitivities but still can be initiated by detonator no. 8 and might be initiated accidentally. PS/BCHMX did not show any reaction under the influence of the impact and friction stimuli. This sample should be quite safe during handling and transportation without risk. Consequently, fabricated PS/BCHMX nanofibrous could be considered a candidate tool to deal with highly sensitive explosives safely and securely for training purposes.

### Thermal behaviors for PS/RDX nanofiber and their raw materials

**Fig. 7:**
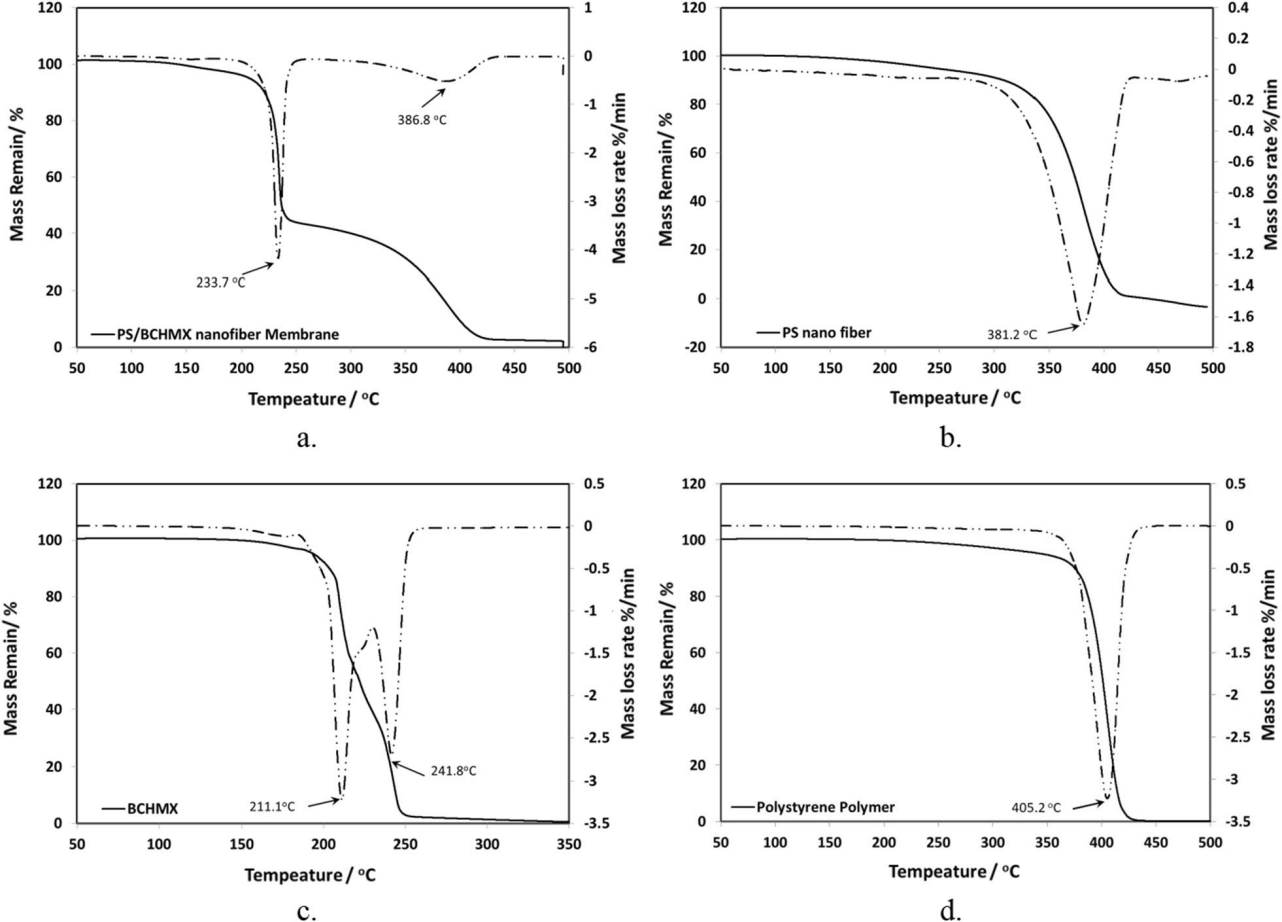
Non-isothermal TG/DTG of **a**. PS/BCHMX Nanofiber **b**. PS nanofiber **c**. Pure BCHMX **d**. polystyrene Polymer at a heating rate of 10 K min^−1^

The TG and DTG curves of PS/BCHMX nanofiber, PS nanofiber, PS Polymer, and pure BCHMX at a heating rate of 10 K min^−1^ in a dynamic nitrogen environment are presented in Fig. 7. In addition, the thermal behavior data of the studied samples are presented in Table 2. Regarding the given data, the decomposition of BCHMX in dynamic nitrogen could be considered as two consecutive steps, while there is only one step for PS nanofiber. Interestingly, PS/BCHMX nanofiber has a two-stage decomposition: the first stage, in the temperature range between about 225.0 to 240.7 °C, in which the mass-loss rate of about 57.9%. The thermal decomposition in this stage was mainly the decomposition of the BCHMX and represents the actual weight of the BCHMX in the studied PS/BCHMX nanofiber membrane. Regarding this stage; the two steps of BCHMX decomposition were changed to a one-step decomposition peak however its maximum peak temperature shift to 233.7 °C, which delivers an increase in its stability due to the well covering of the PS to the BCHMX as was clear in the SEM image in Fig. 3. This confirmation has also represented the increase in the onset temperature of the PS/BCHMX nanofiber compared with the pure BCHMX with a shift reach of 22.9 °C.

**Table 2:** Results of non-isothermal TG/DTG of the PS/BCHMX nanofiber membrane, polystyrene membrane, and pure at a heating rate of 10 K min^−1^

Type	TG curve			DTG curve	
	β/^o^C min^−1^	T_o_/^o^C	Mass loss/%	T_p_/^o^C	T_oe_/^o^C
Polystyrene nano-fiber membrane	10	335.1	99.5	381.2	416.7
Polystyrene polymer		377.9	99.9	405.2	421.7
PS/BCHMX (Peak I)		225.0	57.9	233.7	240.7
PS/BCHMX (Peak II)		309.8	40.9	386.8	424.5
BCHMX (Peak I)		202.1	53.3	211.1	217.8
BCHMX (Peak II)		233.6	45.3	241.8	250.7

The second stage of PS/BCHMX was for the PS nanofiber decomposition, its thermal data was changed from the pure nanofiber membrane see (Fig. 7b). The thermal decomposition is located in the temperature range between about 309.8 and 424.5 °C, which was the main stage of PS nanofiber decomposition with a mass loss of 40.9% with the whole thermal decomposition process of the PS/BCHMX nanofiber membrane. The thermal decomposition of PS/BCHMX nanofiber ended when the temperature was over 500 °C, and the corresponding maximum mass loss rate was about 98.9%. It is obvious, that BCHMX has also a significant effect on the PS polymer by changing its thermal stability by starting the onset temperature from 335.1 to 309.8 °C however, the maximum peak decomposition temperature was shifted from 381.2 to 386.8 °C by 5.6 degrees. Another obvious effect is the width of the decomposition peak of the polystyrene in the presence of BCHMX in the nanofiber which might be connected to the BCHMX decomposition effect inside the fiber and delivering a residue after the thermal decomposition. For DTG of PS polymer, it has thermal decomposition of one stage the same as in the PS nanofiber however there is a certain significant shift in the decomposition data 42.8 °C, 24.0 °C, and 5.0 °C for the onset temperature, the maximum peak temperature, and the end temperature respectively. As was mentioned in Sect. 3.2 the PS nanofiber was changed from the amorphous PS polymer to the crystalline after using the electrospinning which has a tangible change in the thermal behavior data also.

## Conclusions

Using the electrospinning technique with the optimum condition leads to the successful preparation of PS/BCHMX submicron fiber. The SEM images confirmed the complete coverage of BCHMX crystals by PS nanofibers with a size of 200–460 nm. The morphology of the PS submicron-fiber membrane from the amorphous to the crystalline phase was observed by the XRD spectrum due to the effect of the electrospinning preparation conditions. The thermal study confirms the actual percentage of the BCHMX in the PS/BCHMX nanofiber as 57.9%. The thermal decomposition of the PS/BCHMX nanofiber was in two stages with the disappearance of the two consecutive steps of BCHMX decomposition to only a one-step decomposition peak. The PS nanofiber was changed from the amorphous PS polymer to the crystalline state and the thermal behavior data were also changed. The PS nanofibers act as an inhibitor for BCHMX and decreased its sensitivities in addition to their effect on the onset decomposition temperature of BCHMX. The synthesized PS/BCHMX nanofiber is insensitive to different mechanical external stimuli which candidate the electrospinning technique as a tool to deal with highly sensitive explosives safely and securely for training purposes. The study of other high explosives in different fibrous membrane should be interesting topic for future researches.

## Data Availability

Adequate and clear descriptions of the applied materials and tools are provided in the Experimental section of the manuscript. In addition, the obtained data is justified by mentioning the figures and tables in the manuscript.
